# First Successful Liver-Alone Transplantation for TERT (Telomerase Reverse Transcriptase)-Telomeropathy-Related Hepatoportal Sclerosis Cirrhosis

**DOI:** 10.7759/cureus.41296

**Published:** 2023-07-03

**Authors:** Uma Mathuram Thiyagarajan, Samuel Lee, AMJ Shapiro

**Affiliations:** 1 Hepatobiliary-Pancreatic Surgery and Transplantation, University of Alberta Hospital, Edmonton, CAN; 2 Gastroenterology, Faculty of Medicine, University of Calgary, Calgary, CAN; 3 Surgery, Medicine and Surgical Oncology, University of Alberta Hospital, Edmonton, CAN

**Keywords:** orthotopic liver transplantation, cirrhosis diagnosis, end-stage liver disease, telomeropathy, hepatoportal sclerosis

## Abstract

Hepatoportal sclerosis is a rare but well-described condition leading to end-stage liver disease. Telomeropathy is a rare genetic disorder which manifests as premature senescence of cells leading to multisystem disease involving bone marrow, lungs and skin. To the best of our knowledge, there is no report of telomeropathy precipitating end-stage liver disease. Our case presented hepatopulmonary syndrome. Herein, we report a successful liver transplantation in a patient who suffered hepatoportal cirrhosis from telomerase reverse transcriptase (TERT)-telomeropathy.

## Introduction

Hepatoportal syndrome (HPRS) is defined by fibrous intimal thickening of the portal vein or its branches culminating in non-cirrhotic portal hypertension [1]. This condition has been ascribed with different labels including idiopathic portal hypertension (IPH), idiopathic non-cirrhotic portal fibrosis and obliterative portal venopathy by the Japanese, American and Indian authors, respectively, previously [2-4]. Confusion remains on the use of this nomenclature, while HPRS was diagnosed and the International Hepatology Informatics Group recommended the term “hepatoportal fibrosis” in 1994 [5]. There have been few reports of liver transplantation in patients with HPRS [6-9], diagnosed only after the explanted liver had undergone a histological examination.

Telomeres are unique ribonucleoprotein enzymes involved during the replication of the cell cycle [1]. They add telomeric deoxyribonucleoprotein (DNA) repeats to compensate for the loss occurring in each cell replication cycle [10]. Telomerases consist of telomerase RNA and protein subunit telomerase reverse transcriptase (TERT), together forming a catalytic core of holoenzyme [11-14].

Telomere shortening is the hallmark of serial cell division; progressive cell replication leads to shortening at each division ultimately resulting in cellular senescence. Since 2005, a number of mutations in the TERT gene have been characterized, resulting in a myriad of clinical presentations called telomeropathies. In severe forms, they result in bone marrow failure, and pulmonary fibrosis, with substantial morbidity and mortality [15-17]. Due to multisystem involvement by telomeropathy, caring for these patients may be challenging due to the rarity and need for multidisciplinary care. Herein, we report the first successful liver transplantation to our knowledge in a patient with HPRS cirrhosis secondary to TERT-telomeropathy (TERT-T).

## Case presentation

A 32-year-old, Caucasian female presented with a known biopsy-proven HPRS with a background of TERT-T on genetic testing many years ago. She weighed 73 kg and was 1.82 m in height with a body mass index (BMI) of 21.7. Due to the underlying TERT-T, she was also known to have thrombocytopenia with bone marrow biopsy confirming absent hematopoietic elements and complex cytogenetics [46, XX, t(7;22) (q32;q13), -10, +mar11], first presenting at age 13 and followed by the hematology team. Presentation with shortness of breath triggered lung evaluation, and an open lung biopsy was obtained which diagnosed a mild interstitial lung disease from TERT-T. She then developed signs of liver decompensation including hepatic encephalopathy and jaundice and developed hepatopulmonary syndrome (HPS) a few months before she underwent liver transplantation. She required continuous home oxygen therapy from 3 L/min at rest and reached 10 L/min during strenuous physical activity.

The model for end-stage liver disease-sodium (MELD-Na) score was 17 at assessment for liver transplantation. Further echocardiogram with bubble study confirmed intrapulmonary shunting and raised right ventricular systolic pressures (RVSPs). A right heart catheterization revealed mildly elevated RVSP of 26 mmHg with normal pulmonary vascular resistance of 64 dynes/sec/cm^5^, and she was listed for liver transplantation.

Liver transplantation and immunosuppression

A blood group, weight-matched liver from a donor after circulatory death (DCD) became available, and the liver was preserved on an Organox metra (OrganOx Limited, Oxford, United Kingdom) normothermic machine perfusion system (NMP) for 12.5 hours, reassured by a satisfactory ex vivo liver function by clearance of lactate down to 1.2 mmol/L within two hours, and bile production we decided to use the liver. The recipient’s blood investigations including the biochemical profile and complete full blood count are listed in Table 1. Urinalysis and coronavirus disease (COVID-19) screenings were negative.

**Table 1:** Biochemical profile with normal reference values

Parameter	Patient’s Values	Reference Values
Hemoglobin	108 g/L	115-155 g/L
White cell count (WCC)	2.6 × 10^9^ /L	3.5-10.5 × 10^9^/L
Platelets	50 × 10^9^/L	130-380 × 10^9^/L
Bilirubin	47 µmol/L	3-17 µmol/L
Alanine aminotransferase (ALT)	38 IU/L	17-63 IU/L
Aspartate aminotransferase (AST)	43 IU/L	15-34 IU/L
Alkaline phosphatase (ALP)	146 IU/L	40-120 IU/L
International normalized ratio (INR)	1.8	0.9-1.2

**Figure 1:**
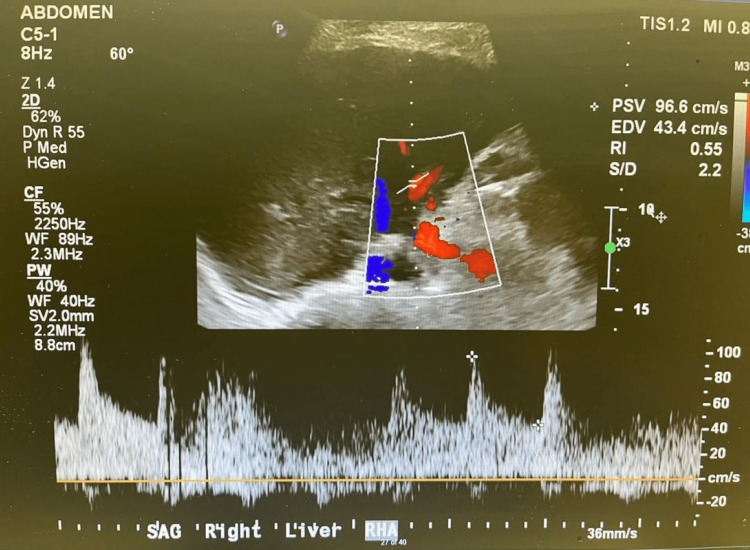
Doppler examination of the right hepatic artery on the first postoperative day

**Figure 2:**
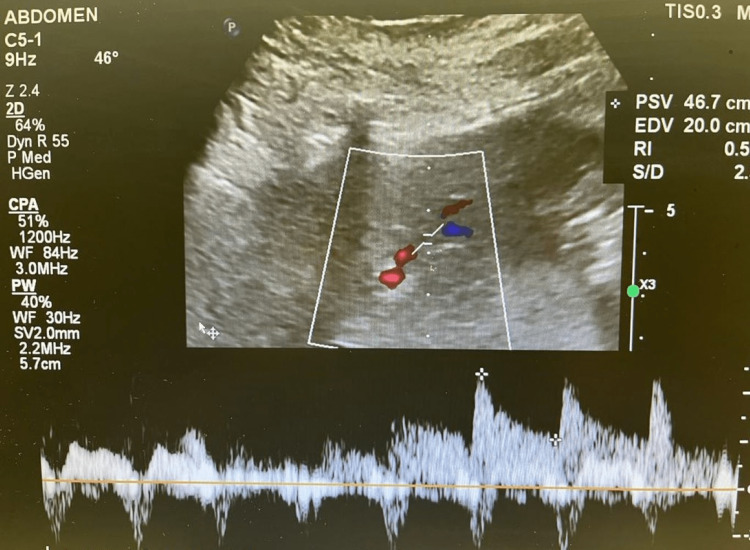
Doppler examination of the left hepatic artery on the first postoperative day

**Figure 3:**
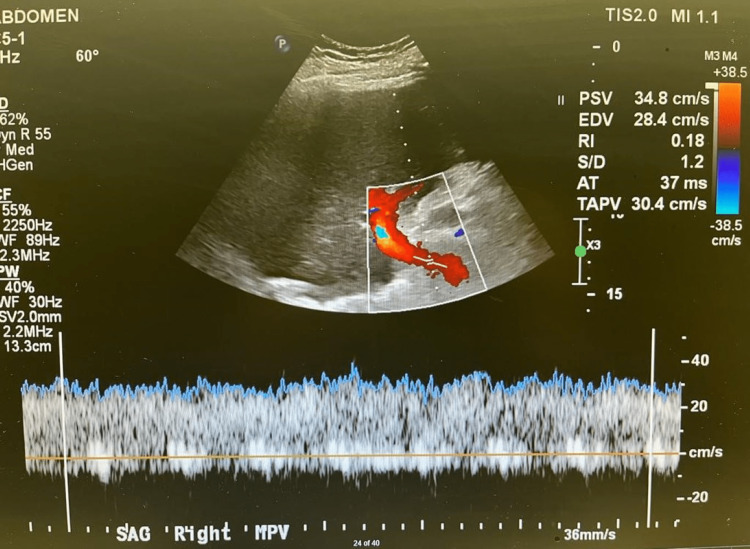
Doppler examination of the right portal vein on the first postoperative day

**Figure 4:**
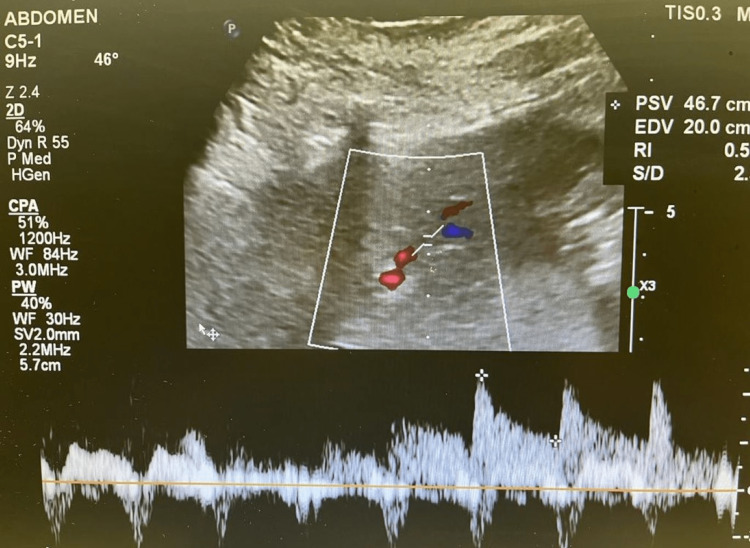
Doppler examination of the left portal vein on the first postoperative day

We proceeded with liver transplantation with caval replacement, single artery and duct-to-duct biliary reconstruction. There was no reperfusion injury syndrome, and the recipient was transferred to the intensive care unit and then moved to the transplant ward on postoperative day 3. A baseline Doppler liver ultrasound that was normal on the first postoperative day showed a satisfactory resistive index of 0.55 and 0.50 at the right and left hepatic artery, respectively, (Figures 1, 2) and portal vein (Figures 3, 4). The immunosuppression regimen consisted of basiliximab induction (Simulect, Novartis Pharmaceuticals Canada Inc, Quebec, Canada) intraoperatively and repeated on day 4. Tacrolimus and mycophenolate mofetil were administered without corticosteroids.

Postoperative challenges

Although she progressed well in the early postoperative, her platelet count started to drop (50 to 20 × 10^9^/L) from the third postoperative day and reached a nadir on day 8 (8 × 10^9^/L). The hemoglobin and white cell count remained within the normal range. Platelet transfusions were given, and prednisone and intravenous immunoglobulin (IVIG) were also added. These agents did not improve the platelet count, and then mycophenolate mofetil and Septra (trimethoprim and sulfamethoxazole combined for *Pneumocystis jirovecii* pneumonia prophylaxis) were discontinued, with subsequent recovery in thrombocytopenia.

Secondly, due to the requirement for pre-existing home oxygen for mild interstitial lung disease/hepatopulmonary syndrome (HPS), she required 15 L/minute but decreased to 5 L/minute within 12 hours post-transplant. No significant pulmonary complications were noted in the postoperative period.

With satisfactory progress, she was discharged on a postoperative day 22, with improving liver graft function with normal renal function and hematological profile. The patient was followed as an outpatient regularly and, at 10 months post-liver transplantation, enjoys improved quality of life. She no longer requires supplemental oxygen at rest, but only during strenuous exercise (treadmill). There are no plans for lung transplantation in her current state. Histopathological examination of the explanted liver confirmed the presence of HPRS again with no evidence of malignancy. Later, the immunosuppression has been tailored to tacrolimus and prednisone in the first three months and then exchanged for cyclosporine monotherapy for hair loss. She developed acute cell-mediated liver transplant rejection during this time, treated with intravenous methylprednisone followed by tapering oral steroid therapy and normalization of function thereafter.

## Discussion

Previous reports describe the HPRS diagnosis made at the histological examination of explanted livers [6-9]. Moreover, most patients are diagnosed with end-stage liver disease (ESLD) secondary to alcohol, autoimmune, hepatitis B or cryptogenic etiology pre-transplant [6-9]. HPRS diagnosis presenting as ESLD is a rare entity. Interestingly, HPRS may present with portal hypertension and preserved liver synthetic function. Hence, the MELD-Na score assessment often underestimates the severity of the liver disease and may disadvantage liver transplantation waiting times.

Lebeer et al. reported lung and liver transplantation in two patients who were found to have telomeropathy in the postoperative period [18]. In their experience, both patients underwent combined transplantation for respiratory failure from interstitial lung disease and cirrhosis. The first patient was diagnosed with HPS and portopulmonary syndrome for which he was treated with liver transplantation initially. Although the lung function improved initially in that reported case, it later deteriorated necessitating lung after liver transplantation at 20 months. Histological examination of the explanted liver showed primary hemochromatosis.

The second patient was initially diagnosed with idiopathic pulmonary fibrosis and morbid obesity, and the preoperative liver biopsy confirmed non-alcoholic steatohepatitis (NASH) cirrhosis. The patient was listed for combined liver and lung transplantation despite a low MELD score of only 9. Once again, the explanted liver confirmed NASH cirrhosis and secondary primary sclerosing cholangitis/hemochromatosis.

There have been no prior reports to our knowledge of TERT-T-associated ESLD presenting as HPRS association with HPS. Our patient had a deranged liver function and hepatic encephalopathy but lacked complications of portal hypertension. This appears unique where ESLD progressed in association with TERC-T.

Due to a complex and multisystem involvement in TERC-T, there are challenges in providing treatment in the setting of ESLD. Where bone marrow manifestations of TERC-T are noted, hematology consultation and modification in immunosuppression may be required. Where HPRS occurs in TERT-T-associated ESLD, potential causes of interstitial lung disease or pulmonary hypertension should be assessed. However, once the HPRS diagnosis is confirmed, liver transplantation may be definitive and life-saving. In our patient, there has been a significant improvement in lung function, specifically the need for oxygen requirement, thus no need for lung transplantation.

## Conclusions

TERT-telomeropathy-related cirrhosis is a unique condition presenting as hepatoportal sclerosis associated with hepatopulmonary syndrome. Isolated liver transplantation offers definitive treatment for TERT-T liver failure and may substantially improve hepatopulmonary syndrome as occurred in our described case. Refractory thrombocytopenia may require modifications of prophylactic or immunosuppressive medications.
